# The New Model of Snail Expression Regulation: The Role of MRTFs in Fast and Slow Endothelial–Mesenchymal Transition

**DOI:** 10.3390/ijms21165875

**Published:** 2020-08-16

**Authors:** Katarzyna Sobierajska, Wojciech M. Ciszewski, Ewa Macierzynska-Piotrowska, Wanda Klopocka, Patrycja Przygodzka, Magdalena Karakula, Karolina Pestka, Marta E. Wawro, Jolanta Niewiarowska

**Affiliations:** 1 Department of Molecular Cell Mechanisms, Medical University of Lodz, 92-215 Lodz, Poland; wojciech.ciszewski@umed.lodz.pl (W.M.C.); emacierzynska@gmail.com (E.M.-P.); magda.karakula@gmail.com (M.K.); karolinapestka89@gmail.com (K.P.); wawro@umed.lodz.pl (M.E.W.); 2 Department of Biochemistry and Cell Biology, Cardinal Stefan Wyszynski University in Warsaw, 01-938 Warsaw, Poland; w.klopocka@uksw.edu.pl; 3 Institute of Medical Biology, PAS, 93-232 Lodz, Poland; pprzygodzka@cbm.pan.pl

**Keywords:** MRTFs, Snail, EndMT

## Abstract

Endothelial–mesenchymal transition (EndMT) is a crucial phenomenon in regulating the development of diseases, including cancer metastasis and fibrotic disorders. The primary regulators of disease development are zinc-finger transcription factors belonging to the Snail family. In this study, we characterized the myocardin-related transcription factor (MRTF)-dependent mechanisms of a human *snail* promoter regulation in TGF-β-stimulated human endothelial cells. Although in silico analysis revealed that the *snail* promoter’s regulatory fragment contains one GCCG and two SP1 motifs that could be occupied by MRTFs, the genetic study confirmed that MRTF binds only to SP1 sites to promote snail expression. The more accurate studies revealed that MRTF-A binds to both SP1 elements, whereas MRTF-B to only one (SP1near). Although we found that each MRTF alone is capable of inducing snail expression, the direct cooperation of these proteins is required to reinforce snail expression and promote the late stages of EndMT within 48 hours. Furthermore, genetic and biochemical analysis revealed that MRTF-B alone could induce the late stage of EndMT. However, it requires a prolonged time. Therefore, we concluded that MRTFs might cause EndMT in a fast- and slow-dependent manner. Based on MRTF-dependent Snail upregulation, we recognized that TGF-β1, as an MRTF-B regulator, is involved in slow EndMT induction, whereas TGF-β2, which altered both MRTF-A and MRTF-B expression, promotes a fast EndMT process.

## 1. Introduction

Endothelial–mesenchymal transition (EndMT) is the process of cellular transdifferentiation regulated under both physiological and pathological conditions, such as fibrosis and cancer [1]. The EndMT-based fibrotic phenotype underlies mainly the following diseases: systemic sclerosis [2,3], pulmonary hypertension [4], diabetic-induced diseases [5,6], sepsis [7], and cerebral cavernous malformations [8]. The crucial role of endothelial cell transdifferentiation was also reported in cancer-associated fibroblast (CAF) formation, the primary regulation of cancer invasion and metastasis [9]. Despite numerous studies, the etiology and molecular mechanisms of these disorders have not been thoroughly elucidated to date.

EndMT is primarily induced by tumor growth factor-β family proteins (TGF-β) [10] through Smad-dependent and Smad-independent pathways (via the Rho family of GTPases) [11,12,13]. EndMT results in the impairment of the cell–cell junction and a spindle-like morphology manifested as increased cell migration and cell contraction abilities. These alterations are accompanied by a loss of endothelial markers expression and the gain of mesenchymal markers [14]. EndMT is also correlated with an increased expression of specific zinc-finger transcription factors, such as Snail and Slug [14,15,16,17,18]; however, their regulation is cell-type dependent.

Rho GTPase activation results in the release and nuclear accumulation of myocardin-related transcription factors (MRTFs). Under physiological conditions, monomeric G-actin forms a stable complex with MRTF, resulting in the sequestration of MRTFs in the cytoplasm. There are two isoforms of MRTFs widely expressed in various tissues: MRTF-A and MRTF-B. The free MRTFs act as co-activators of serum response factor (SFR) via the conserved CArG box DNA element [19,20,21]. TGF-β1-induced EMT in humans and *Canis familiaris* correlated with MRTF-dependent slug overexpression. That change was regulated by Smad binding to the GCCG-like motif in the *slug* promoter [22]. The results of this and other studies and analyses of the regulation of *collagen* expression in lung fibroblasts demonstrated that MRTF-A might act as an SP1 co-activator [22,23]. Schremberg and co-workers demonstrated that MRTF-A regulates cellular transformation into myofibroblasts [24]. Similar studies revealed that the myogenic program is mobilized by a synergy between MRTF and Smad3 [25].

Additionally, Morita and co-workers suggested that MRTFs also determine the regulation of *snail* and *twist* expression. A similar role of MRTFs was confirmed in endothelial cells. In contrast, TGF-β1-stimulated snail overexpression in HK-2 and MDCK cells was regulated through an MRTF-independent pathway [26]. Considering that the regulation of *snail* expression remains lacking, further studies are needed to clarify the involvement of MRTFs in that process

We previously reported that TGF-β2 stimulation of HMEC-1 cells and HUVECs induced late-stage EndMT [27]. It was accompanied by high Snail and MRTFs upregulation and nuclear accumulation of both MRTFs. In contrast, TGF-β1 affected only the MRTF-B translocation and expression and slightly the Snail upregulation. Therefore, in this study, we examined the role of MRTFs in the regulation of snail expression during EndMT. We demonstrated that both MRTFs might regulate Snail expression via the occupation of SP1 sites. We noted that MRTF-A (activated only by TGF-β2) binds to both SP1 sides. In contrast, MRTF-B upregulated by any of TGF-βs is capable of occupying only one of the SP1 sides. TGF-β1, in opposite to TGF-β2, induce only the early stages of EndMT; thus, we proposed that both MRTF isoforms are critical for the induction of the late EndMT stages.

## 2. Results

**Figure 1:**
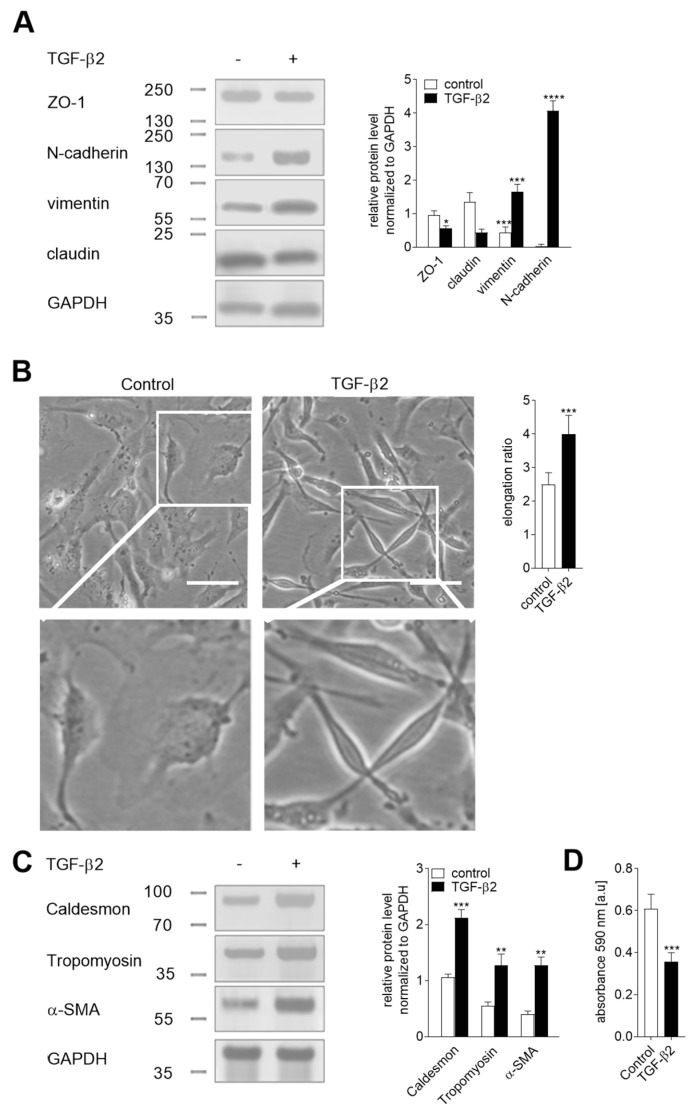
Characteristics of the TGF-β2-induced endothelial–mesenchymal transition (EndMT) in HUVEC. Cells were incubated with TGF-β2 (10 ng/mL) for 48 h or alone. Subsequently, (**A**) the level of EndMT markers was evaluated using Western blot assay with the appropriate antibodies. The background was subtracted and the area for each protein peak was determined. Protein levels were normalized to GAPDH. (**B**) Cell morphology was observed under the microscope. Representative images are shown and a quantitative analysis of the mean of elongation ratio from three independent experiments was performed in Meta^®^Morph. Bars, 30 μm. (**C**) Contraction markers were determined by Western blot analysis. The relative protein level was quantified using scan densitometry. The background was subtracted and the area for each protein peak was determined. Protein levels were normalized to GAPDH. Subsequently, (**D**) the adhesion properties to collagen I was analyzed. Cells were placed on a collagen I pre-coated well for 1 h to adhere. After washing, the cells were stained, and the total cell-associated protein was determined using a microplate reader at 595 nm. The results are presented as the means ± SD (*N* = 3); * *p* < 0.05, ** *p* < 0.01, *** *p* < 0.001, **** *p* < 0.0005.

We previously observed that MRTF-A and MRTF-B are involved in the regulation of EndMT in HMEC-1 cells. We assumed that the cooperation of both isoforms was essential for the induction of the late EndMT stages [27]. To analyze the functions of the MRTFs in EndMT, we utilized HMEC-1 cells, which have been characterized in a previous study as an EndMT model [27]. Additionally, we used HUVECs that undergo EndMT in response to TGF-β stimulation [28]. First, we characterized EndMT in the HUVECs after stimulation with TGF-β2 for 48 h. We observed the decreased expression of endothelial (1.7- and 3-fold changes of ZO-1 and claudin, respectively) and increased mesenchymal (3.7-fold of change of vimentin and appear high expression of N-cadherin) cell markers (Figure 1A). Next, microscopy analysis revealed an approximately 60% elongation of the TGF-β-treated HUVECs that correlated with the overexpression of caldesmon, tropomyosin, and α-SMA (2.0-, 2.3-, and 3.1-fold increased protein level, respectively) (Figure 1B,C). Additionally, the behavioral analysis demonstrated a ca. 40% lower cell adhesion to collagen I of the TGF-β2-stimulated cells (Figure 1D).

**Figure 2:**
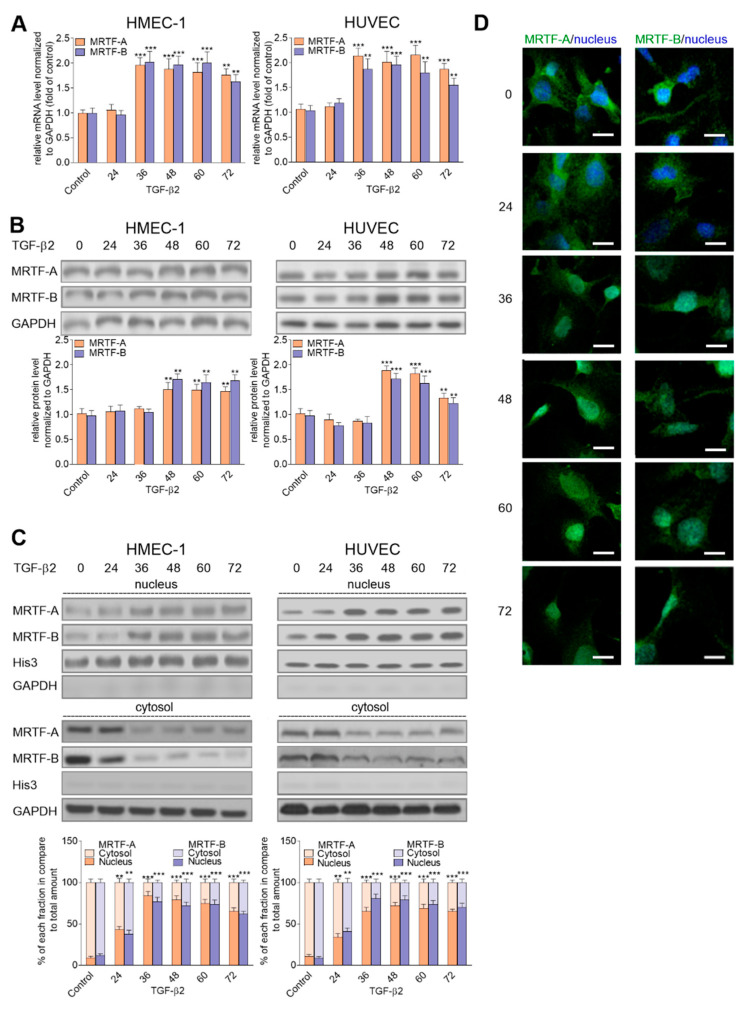
TGF-β2-induced myocardin-related transcription factor (MRTF) upregulation affected their nucleus accumulation. HMEC-1 cells and HUVECs were incubated with TGF-β2 (10 ng/mL) for 24, 36, 48, 60, and 72 h, or alone. Subsequently, (**A**) expression of *MRTF-A* and *MRTF-B* was analyzed by real-time PCR. The protein levels of the MRTFs were determined by Western blot analysis in (**B**) total lysate and (**C**) the nucleus and cytoplasm fraction of the analyzed cells. The background was subtracted and the area for each protein peak was determined. In the analysis of the MRTFs, the translocation MRTF-A or -B level was determined as a % of the total MRTF-A or –B. Protein levels were normalized to GAPDH (total lysate or cytoplasm fraction) or His3 (nuclear fraction). Blots are representative of three independent experiments. (**D**) The localization of MRTF-A and MRTF-B in the cellular compartments were analyzed using a confocal microscope. MRTFs were stained with antibodies conjugated with FITC. The white bar represents a 30 μm length. Representative images are shown. The results are presented as the means ± SD (*N* ≥ 3); ** *p* < 0.01, *** *p* < 0.001.

During EndMT, activated MRTFs are accumulated in the nucleus and act as SRF co-activators to induce the expression of numerous genes. In a previous analysis, we showed an increase in the MRTF-A and MRTF-B protein levels in TGF-β2-stimulated HMEC-1 cells, correlated with the translocation (approximately 90%) of these proteins to the nucleus [27]. The time-dependent analysis revealed that the maximum mRNA (Figure 2A) and protein levels (Figure 2B) of the MRTFs in TGF-β2-stimulated cells were after 36 and 48 h, respectively, in both the analyzed cell lines. In contrast, the increased nuclear translocation of the MRTFs was observed earlier. We detected changes at 24 h after stimulation (Figure 2C,D).

### 2.1. MRTF-Induced Snail Expression in Endothelial Cells

**Figure 3:**
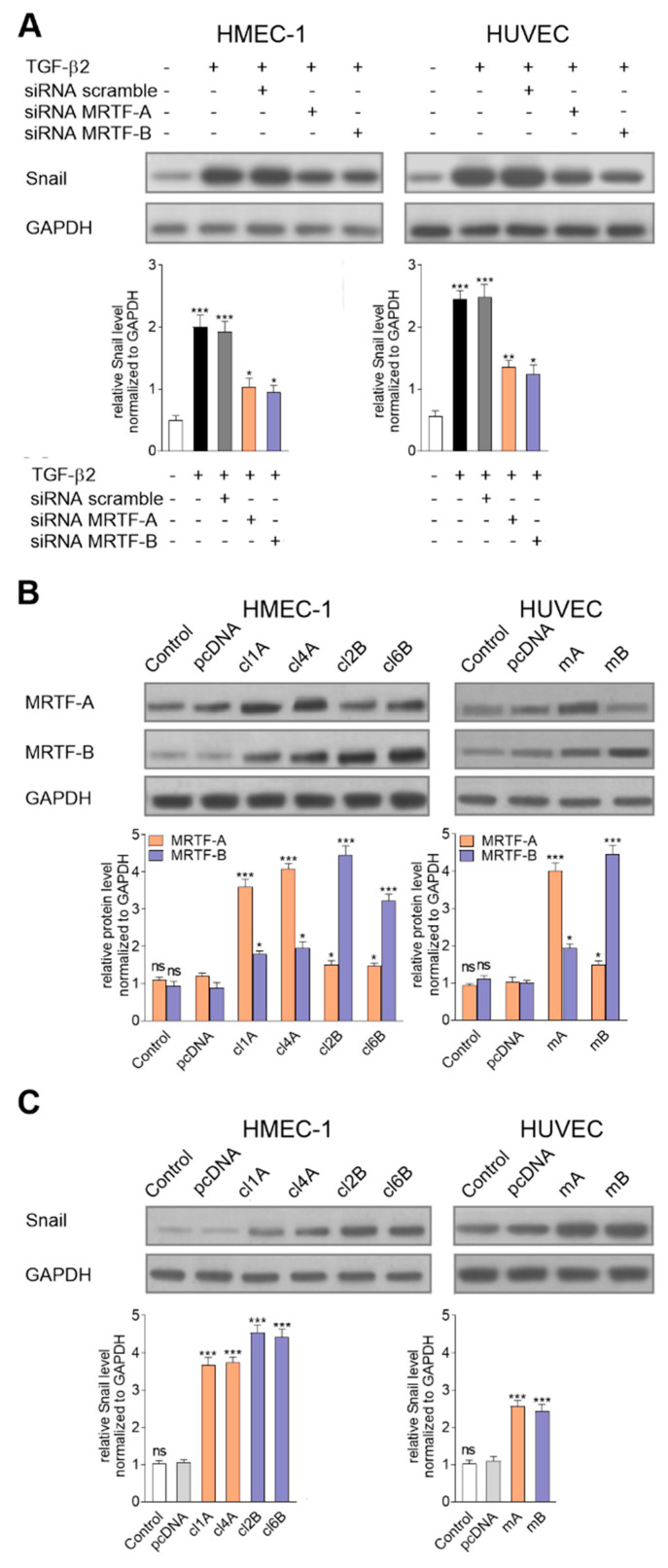
Regulation of the MRTFs caused modulation of *snail* expression. (**A**) Effect of MRTF-A or MRTF-B downregulation using siRNA treatment on the Snail level in TGF-β2-induced cells was analyzed by a Western blot assay. Subsequently, (**B**) MRTF-A (Clone 1—cl1A; Clone 4—cl4A) or MRTF-B (Clone 2—cl2B; Clone 6—cl6B) stably transfected HMEC-1 cells and MRTF-A (mA) or MRTF-B (mB) transiently transfected HUVECs were analyzed. Additionally, the control of the cell transfection clones with only an empty vector (pcDNA) was tested. (**C**) Next, a similar analysis of the Snail level was done. The protein level in each sample was determined using scan densitometry in compared to GAPDH (as the normalization). Blots are representative of three independent experiments. The results are presented as the means ± SD (*N* ≥ 3); * *p* < 0.05, ** *p* < 0.01, *** *p* < 0.001.

The role of MRTFs in the regulation of *snail* expression was studied using an antisense strategy. The cells were incubated with 50 nM of siRNA (according to preliminary studies, this concentration was the most effective; Figure S1) directed against the mRNA of MRTFs for 24 h followed by 48 h stimulation with TGF-β2. We observed that MRTF-A or MRTF-B silencing in the TGF-β2-stimulated cells resulted in the strong (ca. 2-fold) downregulation of the Snail protein levels in both cell lines (Figure 3A). The modulation caused a partially abrogated EndMT effect on the mesenchymal marker upregulations (vimentin and N-cadherin) and cellular elongation (Figure S2). To assess the ability of the MRTFs to induce *snail* expression, we generated stable clones overexpressing MRTF-A and MRTF-B in HMEC-1 cells and transient clones overexpressing MRTF-A and MRTF-B in HUVECs (Figure S3A,B). For further analysis, we selected two clones of the HMEC-1 cells with the highest levels of MRTF-A (3.6- and 4.1-fold upregulation in Clone 1A and 4A, respectively) or MRTF-B (4.5- and 3.2-fold upregulation in 2B and 6B, respectively) (Figure 3B). Additionally, we observed 4.0- and 4,5-fold upregulation in MRTF-A and MRTF-B, respectively, in transiently transfected HUVECs (Figure 3B). The specificity of the applied antibodies in the analyses of the MRTF clones was also tested by Western blots (Figure 1A). Interestingly, we showed that overexpression of one MRTF resulted in the other one’s upregulation. We revealed that MRTF-A or MRTF-B overexpression resulted in a significant increase in Snail protein, with 3.7- and 4.6-fold increases, respectively, in HMEC-1, and 2.6- and 2.4-fold increases, respectively, in HUVECs (Figure 3C).

### 2.2. MRTF Overexpression Induces a Mesenchymal-Like Phenotype in Endothelial Cells

**Figure 4:**
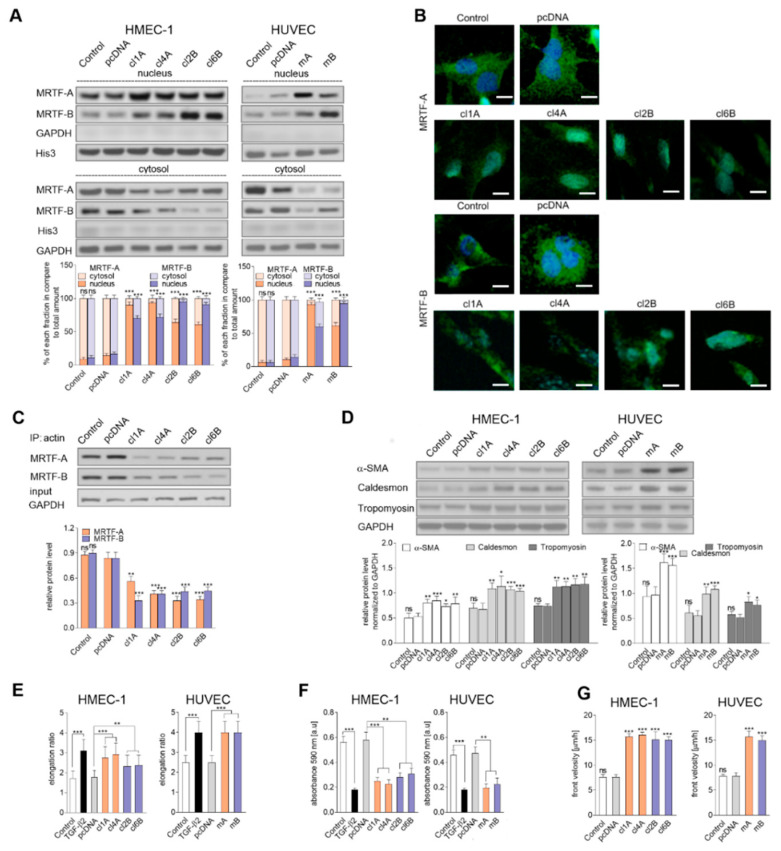
MRTF overexpression resulted in their activation, nuclear accumulation, and induction contraction and adhesion ability. (**A**) MRTF-A and MRTF-B localization were analyzed by Western blotting of the cytosol and nucleus fractions isolated from transfected cells. (**B**) The localization of MRTF-A and MRTF-B in the cellular compartments were analyzed using a confocal microscope. MRTFs were stained with antibodies conjugated with FITC. The white bar represents a 30 μm length. Representative images are shown. (**C**) Additionally, an IP assay of the cytosol fraction protein was applied. The MRTFs that interacted with the actin was precipitated with mouse anti-actin protein and subsequently analyzed by a Western blot assay with anti-rabbit MRTF-A or MRTF-B antibodies. (**D**) Contraction markers, such as α-SMA, caldesmon, and tropomyosin, were determined by Western blot analysis. For (**A**–**D**), the protein level in each sample was determined using scan densitometry. The results were normalized to GAPDH (cytosol and a nuclear fraction or total lysates) or Histone3 (His3, cytosol and nucleus fraction), or total the MRTFs (IP assay). Blots are representative of three independent experiments. The results are presented as the means ± SD (*N* = 3); * *p* < 0.05, ** *p* < 0.01, *** *p* < 0.001. (**E**) Changing in the cell morphology was observed under the microscope. Representative images are shown in the supplementary data (Figure S5) and a quantitative analysis of mean of the elongation ratio ((*N* = 3); ** *p* < 0.01, *** *p* < 0.001) was performed in Meta^®^Morph. Bars, 30 μm. (**F**) Subsequently, adhesion properties to collagen I was analyzed. Cells were placed on the collagen I pre-coated well for 1 h to adhere. After washing, the cells were stained, and the total cell-associated protein was determined using a microplate reader at 595 nm. The results are presented as the means ± SD (*N* = 5); ** *p* < 0.01, *** *p* < 0.001. (**G**) The effect of MRTF-A- or MRTF-B-overexpression on cell movement by the wound healing assay was studied. An appropriate cell number was seeded on 12-well plates pre-coated with collagen 1 (Coll). A total of 48 h later (after 4 h starvation in an FBS-free medium), a monolayer of confluent cells was wounded and images thereof were captured at the following times: 0, 2, 4, 6, 8, 10, 12, and 24 h. The results are given as the standard error of the mean of the cell front velocity ± SD (*N* = 4); *** *p* < 0.001.

Next, analysis of the cell fractions revealed the nuclear accumulation of the MRTFs in MRTF-overexpressing cells (Figure 4A). Similar results were detected using the confocal microscope, where the MRTFs were mainly localized in the nucleus (Figure 4B). We observed the decrease in the MRTF levels in the cytoplasmic fraction. That process correlated with a reduction in the MRTF pool that interacted with G-actin in the MRTF-overexpressing cells (Figure 4C, Figure S4). This finding suggested that free MRTFs are translocated into the nucleus. Next, we examined whether the overexpression of the MRTFs is involved in the behavior of the endothelial cells. First, we observed higher α-SMA levels and the characteristic increased expression of contraction ability proteins, such as caldesmon and tropomyosin (Figure 4D). Similar changes were detected in both cell lines, ranging from 1.6- to 1.7-fold, 1.6- to 1.8-fold, and 1.31- to 1.53-fold for α-SMA, caldesmon, and tropomyosin, respectively. Moreover, we detected a slight elongation (Figure 4E) and ca. 2-fold decreased cell adhesion to collagen I (Figure 4F) in MRTF-overexpressing cells. Next, the investigation of wound healing showed a 2-times increased cell movement in MRTF-upregulated cells (Figure 4G).

### 2.3. Identification of Potential Regulatory Motifs for MRTFs in Snail Promoter

**Figure 5:**
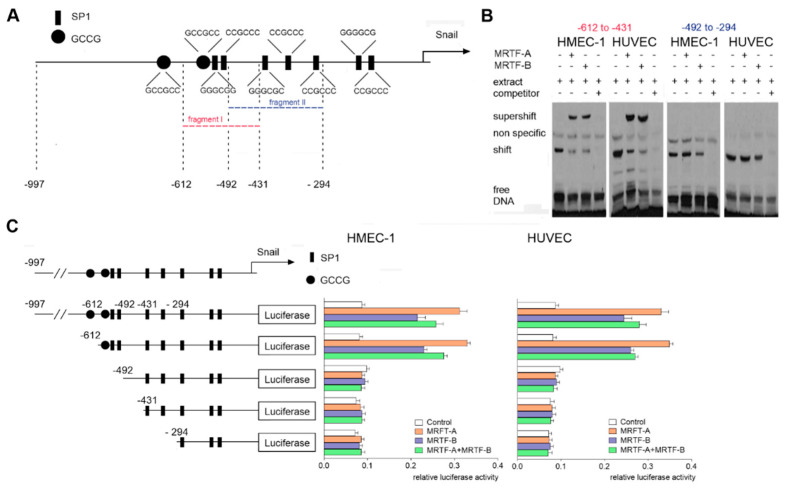
Binding of the MRTFs to the −612/−431 sequence of the human *snail* promoter. (**A**) Schematic map of the *snail* promoter with potential activation sites for the MRTFs. (**B**) Nuclear extracts prepared from the HMEC-1 cells or HUVECs were used for the EMSA assay. For competition experiments, unlabeled competitor oligonucleotides in 200-fold molar excess were used. For the supershift experiments, the rabbit monoclonal anti-MRTF-A or anti-MRTF-B antibodies were used. (**C**) Snail luciferase constructs were studied in MRTF-A-, MRTF-B-, or MRTF-A–MRTF-B-overexpressed HMEC-1 cells or HUVECs. The following constructs were used: −997/90, −612/90, −492/90, −431/90, and −294/90. Results are given as the standard error of the mean of the relative luciferase activity (*N* = 3).

We provided evidence that Snail upregulation is dependent on MRTFs. A similar observation was previously reported, but without further detailed analysis [22]. Thus, we assessed the direct influence of MRTFs on *snail* expression in endothelial cells. The in silico analysis of the human *snail* promoter structure revealed the presence of two fragments potentially regulated through MRTFs (from −492 to −294 and from −612 to −431 bp downstream of the transcriptional start site) (Figure 5A). Further analysis using an Electrophoretic Mobility Shift Assay (EMSA) demonstrated that only the first of the analyzed fragments interacted with the MRTFs (Figure 5B). Indeed, Snail probes were supershifted by the anti-MRTF-A or anti-MRTF-B antibody (Lane 2, 3), thereby demonstrating the presence of a specific binding site for MRTF-A and MRTF-B on the *snail* promoter. Additionally, to confirm the observed results, a series of 5′ promoter deletion mutants of the *snail* gene were prepared. Transfection of the cells with the *snail* promoter construct resulted in a ca. 3-fold induction of the promoter activity (Figure 5C). Similar high activity was observed in the construct started from −612. In contrast, the shorter analyzed sequences starting from −492 had as low a transcriptional activity as the cells transfected with the promoter-less vectors. Thus, these analyses indicated that the MRTF-response elements are between −612 and −492.

### 2.4. MRTFs Regulate of Snail Expression via SP1 Sites

**Figure 6:**
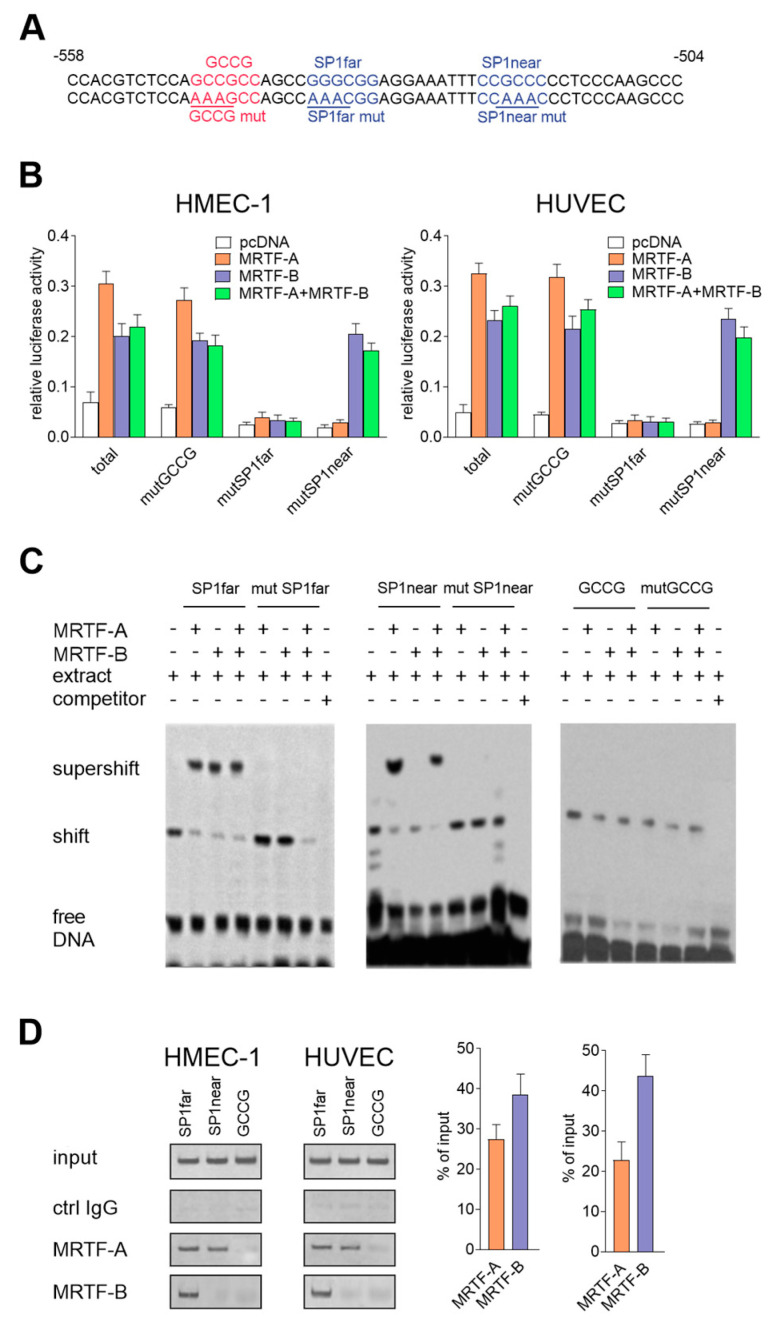
Role of the SP1 motifs in MRTFs-dependent *snail* expression regulation. (**A**) Sequences of mutated GCCG- and SP1-binding sites. Comparison of SP1 sequence localization in the mammalian *snail* promoter (**B**). Snail luciferase mutated constructs were studied in MRTF-A-, MRTF-B-, or MRTF-A–MRTF-B-overexpressed HMEC-1 cells or HUVECs. The constructs with mutGCCG, mutSP1far, and mutSP1near were mutated as indicated in the figure. The results are given as the standard error of the mean of the relative luciferase activity (*N* = 3). (**C**) Nuclear extracts prepared from the HMEC-1 cells were used for the EMSA assay. For competition experiments, unlabeled competitor oligonucleotides, wild-type, or mutated (mutGCCG, mutSP1far, mutSP1near) oligonucleotides were added. For the supershift experiments, the rabbit monoclonal anti-MRTF-A or anti-MRTF-B antibodies and isotype control were used. (**D**) The chip assay was performed in HMEC-1 cells. Chromatin was fragmented and immunoprecipitated with normal rabbit IgG, anti-MRTF-A, and anti-MRTF-B antibodies. Immunoprecipitated DNA was amplified by PCR and separated on an agarose gel and visualized. The representative inverted gel images are shown. The results are presented as the means ± SD (*N* = 3).

The recognized regulatory motif for the MRTFs has three putative binding sequences: two sequences for SP1 (SP1near: from −536 to −531; and SP1far: from −521 to −516) and one sequence for Smad (GCCG box-like motif: from −546 to −541) (Figure 5A). Additionally, the computational analysis revealed that the SP1 sites are preserved among mammalian species. To analyze the functional importance of these putative sites for the MRTFs, we mutated the consensus response elements in all three sites (Figure 6A). Mutational analysis revealed that both SP1 motifs were significant for the MRTF-A-dependent promoter activity, whereas the MRTF-B regulated the *snail* promoter activity by SP1far (Figure 6B). Mutation in the GCCG box-like motif did not affect the promoter activity in response to the MRTFs. Furthermore, the *snail* promoter activation by the MRTFs via the SP1 sites was confirmed using EMSA (Figure 6C) and ChIP assays (Figure 6D). We observed that MRTF-A binds to both SP1 sides, whereas MRTF-B only binds to SP1far. Interestingly, MRTF-B bound with a higher affinity to the SP1far site than MRTF-A (Figure 6D).

### 2.5. Direct Cooperativity of MRTF-A and MRTF-B Is Required for Regulation of Snail Promoter Activity in EndMT

**Figure 7:**
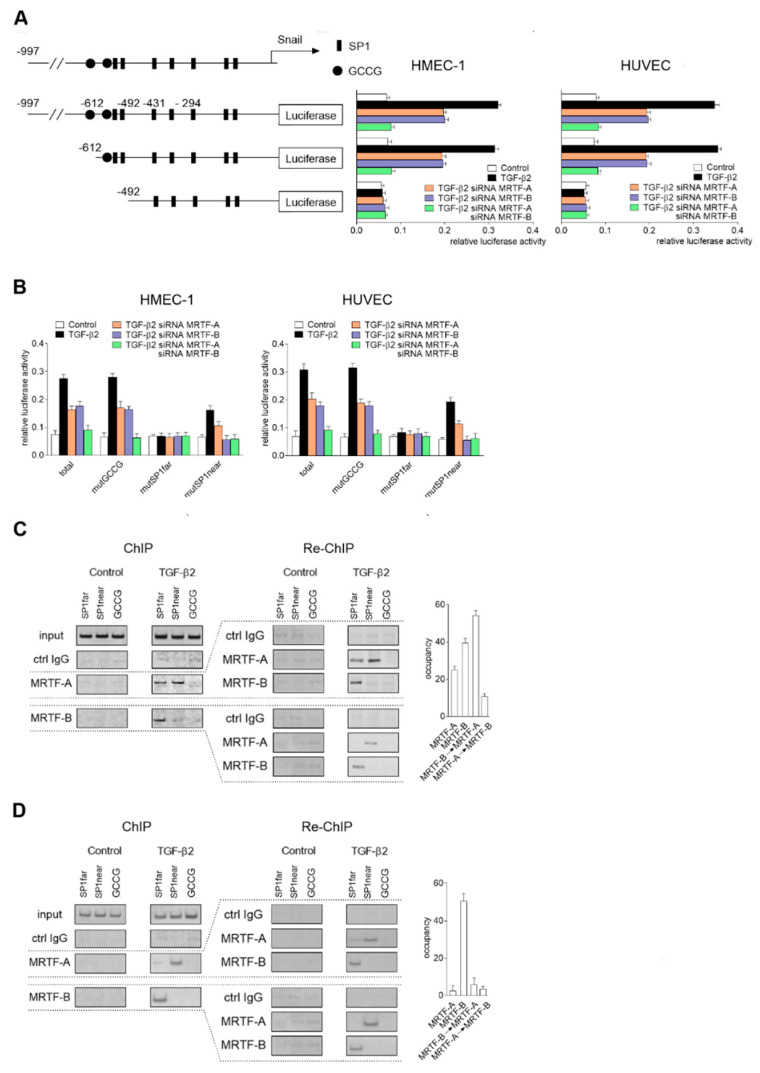
Direct MRTF-A and MRTF-B cooperation is required for complete regulation of *snail* expression in TGF-β2-induced EndMT. HMEC-1 cells and HUVECs were incubated with TGF-β2 (10 ng/mL) for 48 h. As the control, non-stimulated cells were used. Subsequently, (**A**) Snail luciferase constructs were studied in MRTF-A-, MRTF-B-, or both MRTFs silenced (MRTF-A–MRTF-B-silenced) HMEC-1 cells or HUVECs. The following constructs were used: −997/90, −612/90, and −492/90. (**B**) Snail luciferase constructs with mutations in CGGC or SP1 fragments were analyzed in MRTF-A-, MRTF-B-, or MRTF-A–MRTF-B-silenced HMEC-1 cells or HUVECs. The constructs with mutGCCG, mutSP1far, and mutSP1near were made as indicated previously. (**C**) HMEC-1 cells were incubated with TGF-β2 or TGF-β1 (10 ng/mL) for 24, 48, 60, and 72 h. As the control, non-induced cells were applied. Subsequently, the Snail luciferase construct (-997/90) was studied in MRTF-A-, MRTF-B-, or MRTF-A–MRTF-B-silenced HMEC-1 cells. For (**A**) and (**B**), results are given as the standard error of the mean of the relative luciferase activity (*N* = 3). Then, a chip assay was performed in the HMEC-1 cells incubated with TGF-β2 (10 ng/mL) for 48 (**C**) or 72 h (**D**). Chromatin was fragmented and immunoprecipitated with normal rabbit IgG, anti-MRTF-A, and anti-MRTF-B antibodies. For the Re-Chip assay, immunoprecipitated DNA was diluted 10 times and re-immunoprecipitated with normal rabbit IgG, anti-MRTF-A, and anti-MRTF-B antibodies. Immunoprecipitated and re-immunoprecipitated DNA was amplified by PCR and separated on an agarose gel and visualized. Representative inverted gel images are shown. The results are presented as the means ± SD (*N* = 3).

We observed that both the MRTF isoforms are required to induce Snail expression. To elucidate the nature of their interaction in the regulation of *snail* promoter activity, we analyzed the role of MRTF-A and MRTF-B in the regulation of Snail expression in TGF-β2-stimulated cells. The promoter activity after TGF-β2 stimulation remained at the same level as in the MRTF-overexpressing cells (Figure 7A and Figure 5C). Simultaneously, the TGF-β2-dependent transcriptional activity decreased almost two times compared to the activity of the TGF-β2-non-treated cells when the protein level of MRTF-A or MRTF-B was silenced (Figure 7A). It is supposed that both the MRTFs can induce Snail upregulation.

Additionally, transcriptional activity decreased to the basal level when both the MRTFs were silenced (Figure 7A). Further analysis with mutated consensus response elements in the TGF-β2-stimulated cells confirmed the results obtained for the MRTF-overexpressing cells. We observed that both MRTFs were engaged in TGF-β2-induced activation of the *snail* promoter by the SP1far site, and only MRTF-A by the SP1near site (Figure 7B). Thus, we provided clear evidence that SP1far is concurrently the binding site of both MRTF-A and MRTF-B. Finally, to investigate the interactions between both the MRTF proteins, we analyzed the co-occupancy of MRTF-A and MRTF-B to the SP1far side using quantitative a sequential chromatin immunoprecipitation (SeqChIP) assay according to the Geisberg method [29] with future changes [28]. SeqChIP is the method where protein–DNA complexes isolated from the living cells are subjected to two sequential immunoprecipitations with antibodies specifically recognized as two other proteins that can occupy the same place on the gene promoter. The results of the procedure answer whether two proteins can simultaneously co-occupy a stretch of DNA in vivo [29]. The recovery calculation showed that MRTF-B preferentially binds to the SP1far side, even in the presence of MRTF-A after 48 hours of TGF-β2 stimulation (Figure 7C). Therefore, we concluded that MRTF-B is critical for Snail expression. The calculated SeqChIP efficiency, which is related to the extent of the partial co-occupancy between the two proteins, revealed that CMRTF-B→MRTF-A = 63 and CMRTF-A→MRTF-B = 8. Thus, there is a partial co-occupancy and binding of MRTF-A to the SP1far site that depends on MRTF-B. MRTF-B strongly occupied the SP1far side, whereas MRTF-A only marginally binds to the SP1near element (Figure 7D). In conclusion, these results suggest that both MRTFs might be involved in the regulation of Snail expression and that the direct cooperation of MRTF-A with MRTF-B alternate *snail* promoter activity and, therefore, the speed of the EndMT.

### 2.6. Fast and Slow Endothelial-Mesenchymal Transition

**Figure 8:**
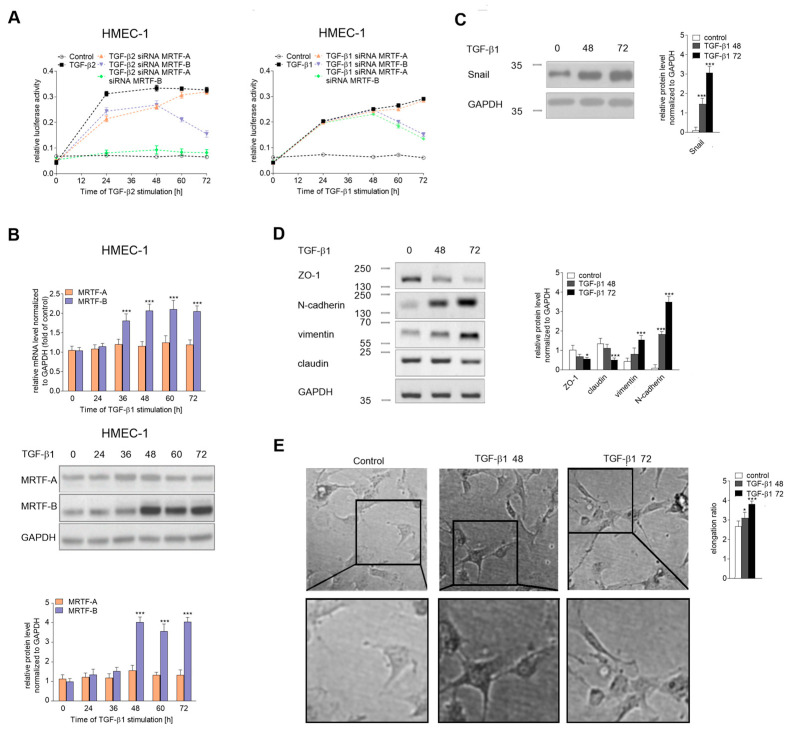
TGF-β1 induced slow EndMT in HMEC-1 cells. (**A**) HMEC-1 cells were incubated with TGF-β2 or TGF-β1 (10 ng/mL) for 24, 48, 60, or 72 h, or alone. Subsequently, the Snail luciferase construct (-997/90) was studied in MRTF-A-, MRTF-B-, or MRTF-A–MRTF-B-silenced HMEC-1 cells. Results are given as the standard error of the mean of the relative luciferase activity (*N* = 3). (**B**) Cells were incubated with TGF-β1 (10 ng/mL) for the indicated time points. Then, the expressions of the MRTFs were analyzed by real-time PCR and the protein level was determined by a Western blot analysis of the total lysate. Cells were incubated with TGF-β1 (10 ng/mL) for 48, 72 h, or alone. Subsequently, (**C**) the level of Snail and (**D**) the levels of EndMT markers were evaluated using Western blot assays with the appropriate antibodies. For the protein level analysis, the background was subtracted and the area for each protein peak was determined. Protein levels were normalized to GAPDH. (**E**) Cell morphology was observed under the microscope. Representative images are shown and quantitative analysis of the mean of elongation ratio from three independent experiments was performed in Meta^®^Morph. Bars, 30 μm. The results are presented as the means ± SD (*N* = 3); *** *p* < 0.001.

We examined the induction of *snail* promoter activity TGF-β2 in a time-dependent manner. We observed that the transcriptional activity of the *snail* promoter in the MRTF-A-silenced cells treated with TGF-β2 was 25% lower than that in TGF-β2-stimulated cells after 24 h (Figure 8A). Moreover, its activity declined after 48 h and reached 40% of the maximum level after 72 h. In contrast, MRTF-B-silenced cells had the same promoter activity as MRTF-A-silenced cells after 24 h. Still, this activity increased in a time-dependent manner and reached a maximum level after 72 h. These findings suggest that MRTF-B might play an essential role in prolonged snail regulation. To confirm our supposition, we performed a similar analysis in TGF-β1-stimulated cells, which resulted in MRTF-B upregulation (both in mRNA and protein level), whereas the MRTF-A level was unchanged (Figure 8B). We demonstrated the TGF-β1 ability to induce the *snail* promoter activity through MRTF-B in a time-dependent manner. However, we observed that in contrast to TGF-β2, where fast stimulation of the *snail* promoter (after 24 hours) was observed, stimulation of the *snail* expression via MRTF-B up to 72 hours of the TGF-β1 induction is required (Figure 8A). Furthermore, to support our observation about the role of MRTF-B in prolonged *snail* regulation, we performed an additional biochemical analysis of time course EndMT induction in the HMEC-1 cells. We observed in a time-dependent manner the TGF-β1 stimulation of the Snail level. A short time of stimulation (shorter than 24 h) has not induced changes in the Snail level (Figure S6). After 72 h, the Snail protein level increased 6-fold (Figure 8C), which was comparable with the Snail level induced with TGF-β2 after 48 h in our previous study [27]. EndMT characterization revealed the time-dependent decreased expression of the endothelial (ZO-1 and claudin) and increased level of mesenchymal (vimentin and N-cadherin) cell markers (Figure 8D). Additionally, microscopy analysis showed approximately 40% elongation of TGF-β1-treated cells after 72 hours (Figure 8E), which correlated with the elongation of the TGF-β2-treated cells within 48 hours in our previous study [27].

## 3. Discussion

The essential role of the Snail family proteins in control of both endothelial– (EndMT) and epithelial–mesenchymal transition (EMT) has been widely supported [15,30,31,32,33]. The Snail family of zinc-finger transcription factors comprises Snail1 (Snail), Snail2 (Slug), and Snail3 (Smuc), which have been implicated in the regulatory processes involved in cell movement, both during embryonic development and invasive and migratory properties during tumor metastasis [34,35,36,37]. Although the role of Snail and Slug in driving cellular transdifferentiation is well-known and broadly accepted, Snail is the primary and crucial factor inducing the initiation of this process [15,38]. Thus, the study of the mechanisms that control *snail* expression required detailed investigations. Recent studies have demonstrated that MRTFs regulate the Snail family protein expression in EMT [22,39]. Moreover, the results obtained in previous studies [12,27,40] showed that the MRTFs might also be involved in such regulation in EndMT. Nevertheless, the detailed mechanisms of *snail* expression regulation through the MRTFs have not been studied at the genetic level, both in EMT and EndMT, to date.

We have previously shown that EndMT induced by TGF-β2 was correlated with both Snail overexpression and MRTF activation in the HMEC-1 cells [27]. In the present study, we also utilized HUVECs undergoing EndMT in response to TGF-β stimulation [28] to confirm that the observed changes are widespread within endothelial cells. 

Firstly, we focused on the analysis of MRTF activation and expression in both EndMT cellular models. We confirmed our previous observation that TGF-β1 could induce only MRTF-B activation and nuclear accumulation [27] in both the analyzed endothelial cells. In contrast, TGF-β2 stimulation resulted in MRTF-A and MRTF-B activation. Our time-dependent biochemical studies revealed that the increased nuclear translocation of the MRTFs was observed earlier than the maximum increase on the transcript and protein levels. We noted that each of the MRTFs’ overexpression resulted in the characteristics for EndMT alteration in cell behavior and the expression profile of endothelial and mesenchymal markers. Previously, it was observed that MRTF-A overexpression correlated with EMT induction [39]. Herein, we found for the first time that overexpression of one MRTF induced upregulation of the second one. Thus, we suppose that upregulation and activation of both proteins are essential for EndMT. From the experiments, we confirmed the hypothesis that silencing the MRTFs caused a partial decrease in the Snail level, one of the primary EndMT regulators. Based on our observation, we have assumed that MRTF-A and MRTF-B regulated snail expression by interaction with a different part of the *snail* promoter.

Based on the EMSA, ChIP, and luciferase reported assays, we demonstrated that the MRTFs are engaged in the regulation of *snail* expression in an EndMT cellular model through binding to the SP1 elements in the *snail* promoter. First, we identified the fragment of the *snail* promoter consisting of a −612/−431 sequence, which was critical for MRTF regulation. The previous studies of the *snail* promoter evidenced that a minimal promoter fragment comprising a −78/+59 sequence was responsible for the constitutive *snail* expression [41]. However, the authors demonstrated that the activity of the promoter was increased in the EMT-induced cells by the regulation elements located in other promoter fragments. Although several regulators are known to induce Snail expression in EMT [42,43,44,45], the mechanisms regulating Snail expression in EndMT have not been studied to date. Our more detailed analysis facilitated the identification of two SP1 elements crucial for the MRTF-dependent regulation of Snail expression: the SP1near and SP1far elements located in −536/−531 and −521/−516 of the *snail* promoter, respectively. We detected that MRTF-A might bind to both of these elements, whereas MRTF-B only bound to the SP1far element. Furthermore, we discovered that the SP1far element is critical for the MRTF-induced *snail* expression. ChIP analysis of the MRTF-overexpressing cells and EndMT-induced cells, as mutations within this element, caused inhibition of *snail* expression to the basal level, as detected in the control endothelial cells. Although MRTF-A might potentially interact with both SP1 elements, the results of the promoter studies indicate that MRTF-A preferentially binds to the SP1near and MRTF-B binds to the SP1far element in the EndMT-induced cells. This finding was confirmed using the Re-Chip analysis, which revealed that MRTF-B occupied the SP1far element in MRTF-A presence, thereby conferring the increased binding of MRTF-B to the SP1far element. Nevertheless, both MRTFs are necessary to regulate the *snail* promoter properly. Although each MRTF was able to induce *snail* expression, we observed that the overexpression of one MRTF leads to the upregulation of the other MRTF. Additionally, the downregulation of any MRTF resulted in a reduction of *snail* promoter activity and a smaller increase in the Snail protein level. Furthermore, we demonstrated that the GCCG element identified in the analyzed *snail* promoter fragment from −546 to −541 was not engaged in Snail regulation. Analysis of *slug* expression regulation in the EMT models showed that MRTF-A binds to the Smad-3 protein. That complex bind to and regulate the activity of the *slug* promoter by the GCCG element [22]. The lack of these results in the present study suggested that the Smad-independent pathways might induce *snail* expression in endothelial cells stimulated through TGF-β to cellular transdifferentiation.

**Figure 9:**
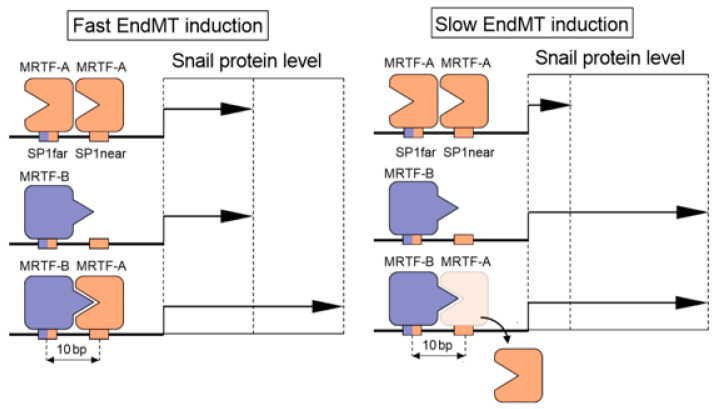
A schematic model of MRTF-induced *snail* expression via SP1 sites in EndMT.3.1. TGF-β stimulates the MRTFs’ upregulation in endothelial cells.

Based on a previous report [27] and the results of the present study, we proposed the theoretical model of the MRTF-dependent regulation of *snail* expression (Figure 9). The results of the present study suggested that there are three different potential mechanisms for the binding of MRTFs to the SP1 sites: (1) MRTF-A occupies SP1far and SP1near; (2) SP1 far is occupied by MRTF-B; and (3) MRTF-B and MRTF-A, respectively, occupy SP1far and SP1near. Although the homogenous occupation of the SP1 sites either by MRTF-A or MRTF-B resulted in the induction of *snail* promoter activity, this mechanism is not responsible for inducing the late stage of EndMT within 48 h. This conclusion is supported by results showing that neither TGF-β2 treatment in MRTF-A-silenced nor MRTF-B-silenced cells were able to induce the late stage of EndMT (likely reflecting the low induction of Snail protein levels). This notion is consistent with previous reports showing that TGF-β1 only induced MRTF-B overexpression [46] and had no significant effects on EndMT within 48 h after induction [47]. Interestingly, considerably higher promoter activity was observed in heterogeneous occupation when MRTF-A and MRTF-B were both bound to the SP1 sites. SP1far and SP1near are separated by complete helical turns (10 bp), enabling the interaction of these proteins and enhancing the promoter activity [48], likely through the stabilization of the interaction between the MRTF-B–MRTF-A–DNA complex. The data obtained from the sequential ChIP assay suggested a mechanism where MRTF-B bind to SP1far and MRTF-A occupied SP1near as the most effective *snail* expression regulator. Additionally, the various activation of snail promoters resulted in higher protein expression sufficient to induce a late stage of the EndMT.

Nevertheless, some studies have suggested that TGF-β1 may induce EndMT in the long-term period (more than 96 h) [11,49]. In the previous studies, we utilized an EndMT model induced by TGF-β in a short time (48 h). However, this time-dependent analysis of the *snail* promoter activated by TGF-β2 showed that MRTF-B alone could activate the promoter to the same level when cooperating with MRTF-A. Still, it is prolonged in time (after 72 h), suggesting the existence of two mechanisms that lead to EndMT induction: fast—requiring the direct cooperativity of MRTF-A and MRTF-B; and slow—depending only on MRTF-B. Additionally, it could be supported by data obtained during a prolonged time of TGF-β2 stimulation (after 72 h) where MRTF-B strongly occupied the SP1far side and the MRTF-A interaction with the SP1near element was only marginal. A similar effect was observed when TGF-β1 stimulation was prolonged to 72 h. To confirm the role of MRTF-B in slow EndMT induction, the HMEC-1 cells were stimulated for 72 h with TGF-β1. Biochemical analysis showed that TGF-β1 is responsible for inducing the late stage of EndMT, confirmed by an increased Snail protein level, alteration of the profile EndMT markers, and cellular elongation, which were accompanied only with a higher MRTF-B protein level.

Summarizing, we introduced a new a model of *snail* expression regulation by MRTFs in the endothelial–mesenchymal transition process. The study reveals the existence of two mechanisms of EndMT induction that depends on the MRTFs’ interaction. Based on the Snail synthesis rate, whose protein is a well-known EndMT stimulator, we proposed that EndMT is induced in a fast- and slow-dependent manner (Figure 9). Fast EndMT observed 48 h after induction is regulated by the cooperation of MRTF-A and MRTF-B with the *snail* promoter. Interaction of MRTF-A–MRTF-A or just MRTF-B with the *snail* promoter induced the Snail upregulation only slightly and cannot induce EndMT within 48 h. However, prolonging the time to 72 h after induction resulted in higher induction of the Snail protein levels required to observed late stages of EndMT. We called these mechanisms slow EndMT induction, and it depends only on the MRTF-B presence over a prolonged time. We recognized that TGF-β1, as an MRTF-B regulator, is involved in slow EndMT induction, whereas TGF-β2, which altered *MRTF-A* and *MRTF-B* expression, promotes a fast EndMT process.

## 4. Materials and Methods 

### 4.1. Reagents

Tissue culture reagents, including MCDB 131 medium, fetal bovine serum, and antibiotics were obtained from Life Technologies (Paisley, UK). cOmplete™, EDTA-free Protease Inhibitor Cocktail was purchased from Roche (Basilea, Switzerland), while TGF-β2 was purchased from R&D (Minneapolis, MN, USA). GelShift Chemiluminescent EMSA Kit, Dual-Luciferase Reporter Assay System was purchased from Promega (Madison, WI, USA). The Enhanced Chemiluminescence (ECL) Western blotting substrate, M-PER Extraction Reagents, NE-PER Nuclear and Cytoplasmic Extraction Kit from Thermo Scientific Pierce (Rockford, IL, USA). X-fect was purchased from Clontech (Mountain View, CA, USA) and siRNA oligonucleotides from Dharmacon (Lafayette, CO, USA). All starters were synthesized by Genomed (Warsaw, Poland). Mouse anti-Snail ((L70G2); #3895) antibody and Epithelial-Mesenchymal Transition (EMT) Antibody Sampler Kit #9782 (rabbit anti-Vimentin D21H3; #5741, anti-N-cadherin D4R1Hm #13116, anti-ZO-1 D7D12 #8193; anti-claudin D5H1D; #13255) were purchased from Cell Signaling Technology (Danvers, MA, USA), and goat anti-mouse and anti-rabbit antibodies conjugated with horseradish peroxidase and rabbit anti-MRTF-A((H-140): sc-32909), anti-MRTF-B (H-53): sc-98989 and mouse anti-GAPDH (G-9; sc-360562) antibodies were purchased from Santa Cruz Biotech (Santa Cruz, CA, USA). Reagents for Western blot assay were obtained from Bio-Rad (Munich, Germany). All other chemicals were purchased from Sigma-Aldrich, including rabbit anti-His3 (#H0164), anti-caldesmon (#SAB4503188), anti-tropomyosin (#AB5441), mouse anti-α-SMA (#A2547), and anti-actin (#AC-74) antibodies.

### 4.2. Cells Line and Culture Conditions

HMEC-1, a gift from Kathryn Kellar, Centers for Disease Control and Prevention, Atlanta, GA, USA, were maintained in MCDB 131 medium supplemented with 10% (*v*/*v*) fetal bovine serum, streptomycin (100 μg/mL), penicillin (100 units/mL), glutamine (2 mM), EGF (10 ng/mL), and hydrocortisone (1 μg/mL). HUVECs were isolated from veins of freshly collected umbilical cords as described elsewhere [50]. Cells isolated from three individual donors were pooled, maintained as HMEC-1 in a medium without hydrocortisone, and Passages 2–5 were used for the experiments. All cells were grown at 37 °C in a humidified atmosphere with 5% CO_2_ and were routinely tested and confirmed as mycoplasma-free. The EndMT development cells were stimulated with TGF-β2 for an appropriate time. The stable MRTF-A or MRTF-B HMEC-1 clones were additionally supplemented with 50 μg/mL of geneticin. In experiments with siRNA, technique oligonucleotides were added for 33 h.

### 4.3. Co-Immunoprecipitation and Western Blot Analysis

Briefly, for the co-immunoprecipitation experiments, 500 μg of protein of cytoplasmic fraction isolated from HMEC-1 cells were taken. Firstly, it was precleared with 30 μL of protein A/G-agarose bead slurry for 2 h at 4 °C to avoid nonspecific binding and incubated with 2 μg of anti-actin antibodies or anti-MRTF-A and MRTF-B antibodies on a rotator overnight at 4 °C. Subsequently, 100 μL of protein A/G-agarose bead slurry was added, and the incubation was continued for another 3 h. The beads were washed three times with PBS, suspended in 2× concentrated SDS-PAGE loading buffer, and boiled for 5 min. Cell lysates and fractions were prepared as previously described [46]. Immunoprecipitates or lysates were separated on 10% Tris-Glycine gels and electrotransferred onto nitrocellulose membranes. Developed films were scanned by an HP Scanjet G4050 scanner and protein bands were quantified using ImageJ software.

### 4.4. Cell Morphology Analysis

Cell morphology was analyzed using a microscope (Olympus, Olympus Optical Co., Ltd., Tokyo, Japan) and the representative images were captured using an Olympus digital camera. Next, the changes in cell morphology were estimated by measuring the elongation ratio (the ratio of the longer to the shorter axis) of the analyzed cells using Meta^®^Morph [51].

### 4.5. RNA Isolation and Real-Time PCR

RNA isolation and purification using TriPure reagent were performed as previously described. The total RNA (1 μg) from cells was reverse transcribed using Moloney Murine Leukemia Virus Reverse Transcriptase and real-time PCR was performed in triplicates using the LightCycler (Roche Diagnostic, Indianapolis, IN, USA). Relative expression was normalized to GAPDH. The following primers were used: Snail (forward 5′-GCTCGAAAGGCCTTCAACTGCAAA-3′ and reverse 5′-AGGCAGAGGACACAGAACCAGAAA-3′), and GAPDH (forward 5′-GAGAGATGATGACCCTTTTGGC-3′ and reverse 5′-CCATCACCA TCTTCCCAGGAGCG-3′).

### 4.6. Confocal Microscopy

As described previously [52], a total of 1 × 10^4^ cells was placed on sterile glass microscope slides and cultured at 37 °C in a humidified atmosphere of 5% CO_2_. After 24 h, the cells were washed with PBS, fixed with 4% formaldehyde in PHEM buffer (60 mM Pipes, pH 6.9, containing 25 mM Hepes, 10 mM EGTA, 4 mM MgCl_2_) supplemented with protease inhibitors (cOmplete™, EDTA-free Protease Inhibitor Cocktail) for 20 min at room temperature, and prepared as described previously [29]. The cells were then washed three times with the PHEM buffer, permeabilized with 0.1% Triton X-100 (*v*/*v*), and blocked with 2% (*v*/*v*) BSA in PHEM buffer for 60 min at room temperature. MRTFs and α-SMA localization were detected by cell staining with antibodies conjugated to fluorescein isothiocyanate (FITC) for 60 min at 37 °C. A confocal laser microscope Leica TCS SP5 system (Leica Microsystems GmbH, Mannheim, Germany) was used for intracellular probe visualization. A series of single 0.2-μm optical sections were collected. The images were scanned at a high resolution (63× oil objective, 1.4 NA).

### 4.7. siRNA Silencing

Cells were transfected with siRNAs targeting human: MRTF-A and/or MRTF-B and the negative control siRNA using X-fect^®^ reagent according to the manufacturer’s instructions. The siRNA treatment was performed at 15 h after TGF-β stimulation, and the appropriate gene transcriptions were silenced for 33 h.

### 4.8. Stable Transfection

The expression vector with MRTF-A (MRTF-A-pcDNA3.1), MRTF-B (MRTF-A-pcDNA3.1), and the empty (pcDNA3.1) vectors (gifts from Muh-Hwa Yang, Ph.D. Institute of Clinical Medicine, National Yang-Ming University, Taipei, Taiwan) were introduced into HMEC-1 cells using the Xfect^®^ reagent according to the manufacturers’ instructions. After 24 h, the cells were maintained in medium supplemented with geneticin (50 μg/mL) for 2 weeks and the geneticin-resistant colonies were subjected to sub-cloning. Expressions of the MRTF-A or MRTF-B protein in the selected clones were analyzed using Western blot assays. Two stably transfected Clones, 1 and 6, of MRTF-A, as well as 2 and 4 of MRTF-B, were taken for further analysis.

### 4.9. Cell Adhesion Assay

Wells of F8 Maxisorp loose Nunc-Immuno^TM^ modules (Nunc^TM^ brand products) were coated with 10 μg/mL of collagen type I in TBS (0.02 mM Tris/HCl, 0.15 mM NaCl, pH 7.5) and the analysis was made as previously described [27]. The total amount of cell-associated protein was determined using a microplate reader at 595 nm (BioKinetics Reader EL340, Bio-Tek Instruments, Winooski, VT, USA).

### 4.10. Wound Healing

Cells grown to confluent on 12-well plates were starved for 4 h in an FBS-free medium and wounded across the cell monolayer by scraping the cells with a 200 μL pipette tip. Subsequently, the cells were treated as previously described [46], and images were captured every 2 h (from 0 to 12 h) and after 24 h by an inverted Nikon phase-contrast microscope (400× magnification) with a digital camera (Olympus IX81, Olympus Optical Co., Ltd., Tokyo, Japan). Migration was quantified using ImageJ software.

### 4.11. Luciferase Reporter Assay

A luciferase reporter assay was performed using a Dual-Luciferase Reporter Assay (Promega). The promoter region for a *snail* (−997/92) or they fragment was amplified by PCR and cloned into the pGL4 vector (Promega). HMEC-1 cells or HUVECs were transfected with the pGL4-snail/promoter together with pQCXIP-B-Myb and pRTK-Luc to normalize the transfection efficiency. A Total of 48 h later, the activities of firefly luciferase and Renilla luciferase were measured using the Dual-Luciferase Reporter Assay System according to manufacturer instruction. Luciferase activities were measured in a Wallac 1420 VICTOR2 Multilabel Counter (PerkinElmer Shelton, CT, USA).

### 4.12. Electrophoretic Mobility Shift Assay

Nuclear extracts were isolated as described in the Western blot method. EMSA experiments were conducted using the GelShift Chemiluminescent EMSA Kit according to the manufacturer’s instructions. For competition experiments, unlabeled competitor oligonucleotides in a 200-fold molar excess were used. For the supershift experiments, the rabbit monoclonal anti-MRTF-A or anti-MRTF-B antibodies were used. The oligonucleotide probe (AACTGAGAAAGAGAAAGACGACA) homologous to the −398/−376 fragment of MMTV promoter region upstream the transcription initiation start was used as a positive control [53] and a non-specific oligonucleotide probe (GCTCAGAACATGTCTAAGCATGCTTCGGCT) deprived of *snail* binding motifs [54] was used as the negative control. After sample separation in polyacrylamide gels and electroblotting, DNA was cross-linked to the membranes under the UV light. The biotin-labeled probes were detected using streptavidin conjugated to horseradish peroxidase, processed for chemiluminescent and visualized using an HP Scanjet G4050 scanner. Protein bands were analyzed using ImageJ software.

### 4.13. Chromatin Immunoprecipitation (ChIP) and Re-ChIP

Chromatin immunoprecipitation (ChIP) experiments were performed using the Pierce Agarose ChIP Kit according to the manufacturer’s instructions. Cells were cross-linked in 1% formaldehyde for 10 min, washed in PBS, and lysed in PBS with 1% SDS. Subsequently, the chromatin was sonicated (Vibracell, BioBlock, Newtown, CT, USA) at an amplitude setting of 50% for 40 seconds on ice to obtain an average length of 400 bp. Next, the cross-linked protein-DNA complexes extracted from cells were preincubated with protein. Agarose beads were added for 1 h to remove non-specific interactions between the protein and beads, centrifuged (14,000 rpm, 5 min 4 °C), and immunoprecipitated with anti-MRTF-A or anti-MRTF-B antibodies overnight at 4 °C. DNA was purified from the immunoprecipitated complexes in a 1% SDS buffer and the 200–250 bp fragments of the larger part of the *snail* gene promoter region. At this point, 10 μL was removed from each sample (except those not used for SeqChIP) for subsequent analysis of the first immunoprecipitation. For the Re-ChIP analysis, the immunoprecipitate was incubated in the regeneration buffer (75 mL PBS/10 mM DTT with 5 mM MgCl_2_) for 2 h in 4 °C. Next, the obtained homogenate was diluted 1:10 in PBS and re-immunoprecipitated with antibodies as described above. The PCR products were separated by electrophoresis in 7% polyacrylamide gels in a Tris-acetate EDTA (TAE) buffer, stained with ethidium bromide, visualized by UV light, and documented using the GelDoc EQ gel documentation system (Bio-Rad, Hercules, CA, USA). Primers were used in real-time PCR: 5′-CCATACAATGAATAGTCCGCATCC and 5′-GAACCTCCACACTAACCTACACC; and 5′-GAGCAGCCCTTAATGACTTG and 5′-CCCAACTCCCTAACTTCCC, respectively. The results of a Re-ChIP experiment of co-occupancy were calculated as previously described [29]. The SeqChIP efficiency (C; in percent) was determined according to the formula C = 100(AB − A)/(A · B − A), where AB represents the fold-enrichment for the sequential ChIP and A and B represent the fold-enrichments for the individual ChIPs. When the order of the IPs was reversed (B ChIP is first, A ChIP is sec), C was calculated according to the formula C = 100(BA − B)/(B · A − B).

### 4.14. Point Mutation

The point mutants we produced using the GeneTailor Site-Directed Mutagenesis System (OriGene Technologies, Rockville, MD, USA) with the appropriate primers propagated in *E. coli*, purified with Wizard Midiprep, and sequenced to confirm the open reading frame.

### 4.15. Computational and Statistical Analysis

Putative transcription factor elements within the *snail* gene promoter were identified using MatInspector software. The alignment of the *snail* promoters from various mammalian species was performed using the CLUSTALW program. Genomic sequences were downloaded from the University of California Santa Cruz genome browser.

All data were collected from at least three independent experiments. Statistical analysis and graphical presentation were performed using GraphPad Prism Software v 8.4 for Windows (San Diego, CA, USA). Statistical significance was evaluated using Student’s t-test or one-way ANOVA followed by post hoc Tukey’s test. Differences between the means were considered to be significant when *p* ≤ 0.05. The data are presented as the means ± standard error unless otherwise stated.

## 5. Conclusions

In summary, the cellular transdifferentiation causing proliferative diseases, such as cancer and fibrosis, might be an attractive target for new therapies. Thus, the understanding of molecular mechanisms regulating EMT or EndMT appears indispensable. The results of the present study provided the first evidence that direct MRTF-A and MRTF-B cooperation is essential in the complete regulation of *snail* expression in EndMT-induced cells. Therefore, the current findings may lead to the development of strategies to manipulate these signals for the therapeutic benefit of cancer or fibrosis treatment.

## Supplementary Materials

Supplementary materials can be found at https://www.mdpi.com/1422-0067/21/16/5875/s1.

## Abbreviations

MRTFs	Myocardin-related transcription factors
TGF-β	Tumor growth factor-β
EndMT	Endothelial–mesenchymal transition
