# Supplementary material for: The New Model of Snail Expression Regulation: The Role of MRTFs in Fast and Slow Endothelial–Mesenchymal Transition
Source: Int J Mol Sci. 2020 Aug 16;21(16):5875. doi: 10.3390/ijms21165875 (PMC7461591; doi:10.3390/ijms21165875)
Supplement: Supplementary file 1 [file ijms-21-05875-s001.pdf]

Figure S1

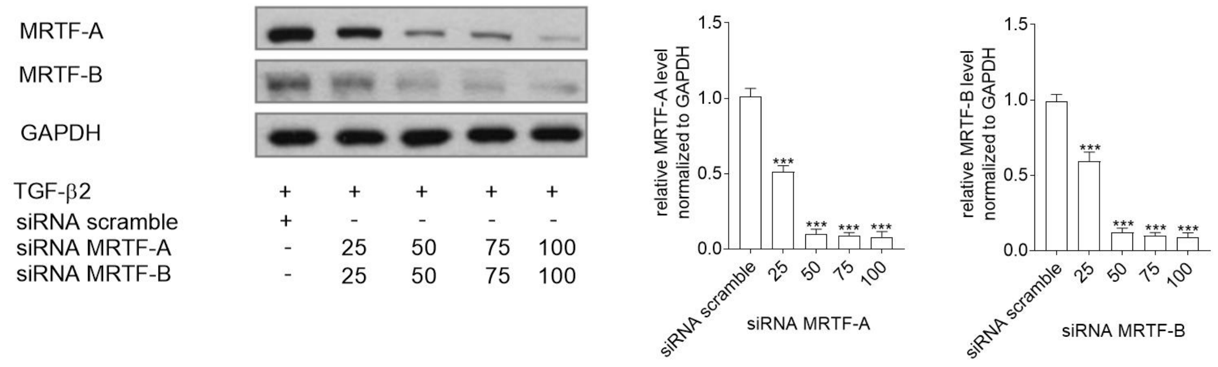

Optimization of siRNA treatment.

The optimal siRNA concentrations for silencing MRTF-A or MRTF-B expression were determined by Western blot assay in cells treated with different concentrations (25, 50, 75, 100 nM) of siRNA specific to appropriate protein. Based on the obtained results, the concentration of 50 nM was chosen for subsequent MRTFs analysis. The relative protein level was quantified using scan densitometry. The background was subtracted, and the area for each protein peak was determined. The protein levels were normalized to GAPDH. The results were given as the means ± SD (N=3); ***p < 0.001. The blots are representative of three independent experiments.

Figure S2

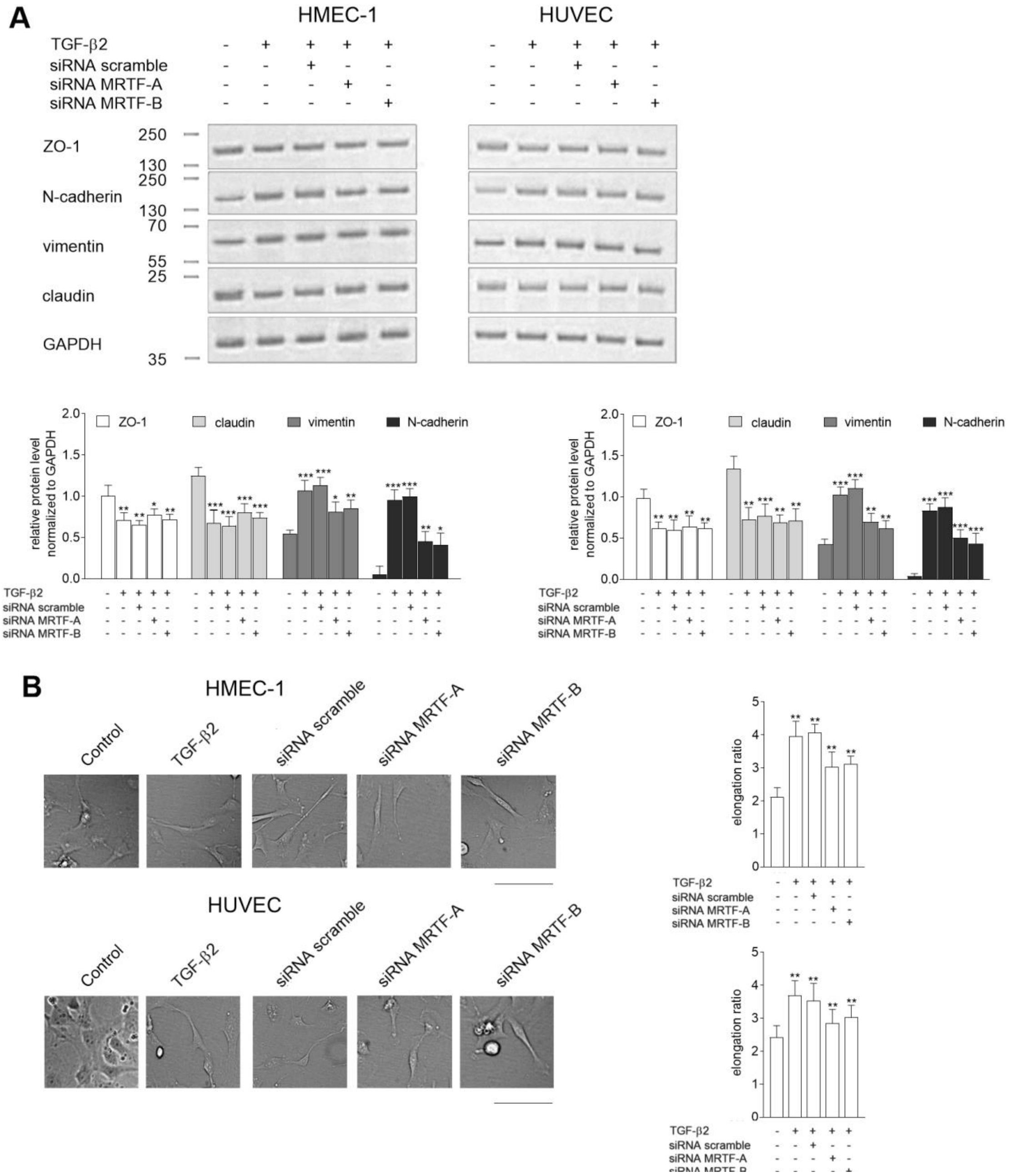

Characterization of EndMT in stimulated cells where MRTF-A or MRTF-B was silenced.

(A) Western blot assays determined the level of endothelial (ZO-1, claudin) and mesenchymal (N-cadherin, vimentin) markers with antibodies recognizing particular proteins in lysates from TGF-β2-stimulated cells (line 2) where MRTF-A (line 3) or MRTF-B (line 4) were silenced. The relative protein level was quantified using scan densitometry. The background was subtracted, and the area for each protein peak was determined. The protein levels were normalized to those of GAPDH. (B) Cell morphology was observed under the microscope. Representative images are shown and quantitative analysis of mean of elongation ratio from three independent experiments was performed in Meta®Morph. Bars, 30 µm.The results were given as the means ± SD (N=3); p<0.05 ** p<0.001 ***p < 0.005. The blots are representative of three independent experiments.

Figure S3

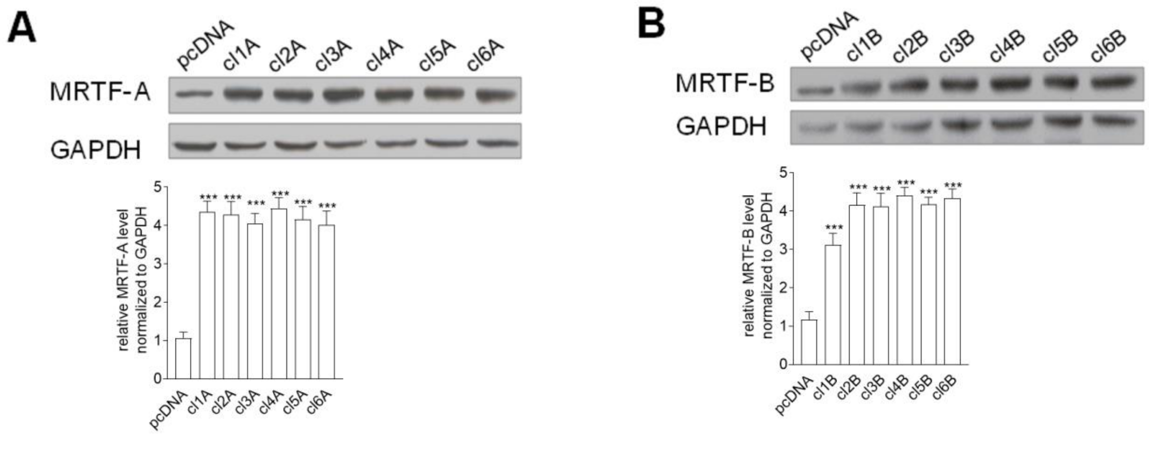

Analyzing of MRTFs expression in HMEC-1 clones.

The expression vector with MRTF-A (MRTF-A-pcDNA3.1), MRTF-B (MRTF-A-pcDNA3.1) and empty (pcDNA3.1) vectors were introduced into HMEC-1 cells using Xfect® reagent according to manufacturers instruction. After 24 hours, cells were maintained in medium supplemented with geneticin (50 μg/ml) for two weeks and the geneticin-resistant colonies were subjected to sub-cloning. Expressions of the MRTF-A or MRTF-B protein were determined by Western blot assay. The relative protein level was quantified using scan densitometry. The background was subtracted, and the area for each protein peak was determined. The protein levels were normalized to GAPDH. The results were given as the means ± SD (N=3); ***$p < 0.001$. The blots are representative of three independent experiments.

Figures S4

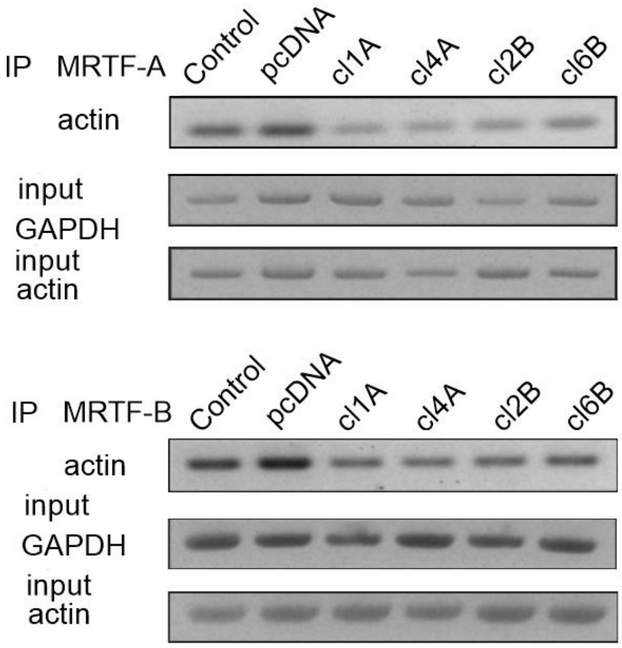

Analyzing of MRTFs-actin interaction

To determine the interaction between MRTF-A and MRTF-B with actin co-immunoprecipitation assay followed by Western blot analysis was performed as described in Material and Methods. Co-immunoprecipitation by MRTF-A or MRTF-B antibodies followed by Western Blot assay with antibodies recognized actin was presented.

Figure S5

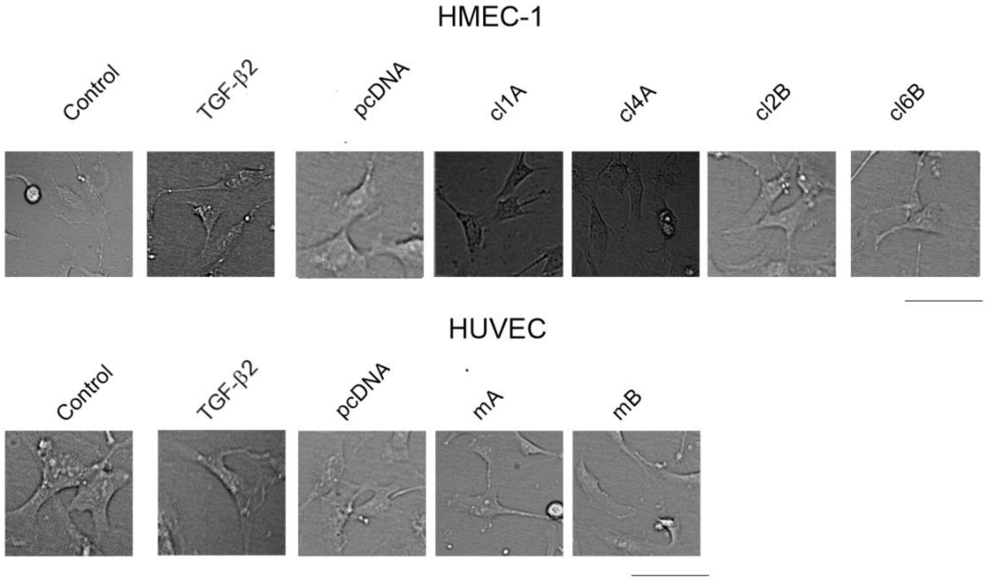

Cell morphology was observed under the microscope. Representative images are shown; Bars, 30 μm.

Figure S6

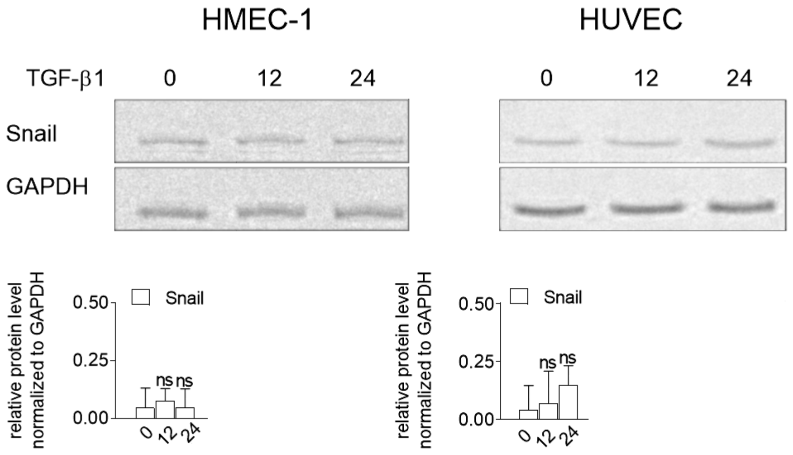

Snail level alteration after a short time of TGF-βs stimulation

Western blot assays determined the Snail level in lysates from TGF-β1 stimulated cells during 12 (line 2) or 24h (line 3). The relative protein level was quantified using scan densitometry. The background was subtracted, and the area for each protein peak was determined. The protein levels were normalized to GAPDH. The results were given as the means ± SD (N=3); ns- non statistical. The blots are representative of three independent experiments.
